# Distinct role of interleukin-6 and tumor necrosis factor receptor-1 in oval cell- mediated liver regeneration and inflammation-associated hepatocarcinogenesis

**DOI:** 10.18632/oncotarget.11365

**Published:** 2016-08-18

**Authors:** Tong Ji, Gaofeng Li, Jiang Chen, Jie Zhao, Xi Li, Hui Lin, Xiujun Cai, Yong Cang

**Affiliations:** ^1^ Life Sciences Institute and Innovation Center for Cell Signaling Network, Zhejiang University, Hangzhou, Zhejiang, 310058, China; ^2^ Department of General Surgery, Sir Run Run Shaw Hospital, College of Medicine, Zhejiang University, Hangzhou, Zhejiang, 310058, China; ^3^ Oncology Business Unit and Innovation Center for Cell Signaling Network, WuXi AppTec Co., Ltd., Shanghai 200131, China

**Keywords:** IL6, TNFR1, oval cell, hepatocellular carcinoma, NK cell

## Abstract

Interleukin 6 (IL6), tumor necrosis factor α (TNFα) and TNF receptor-1(TNFR1) have been shown to involve in oval cell proliferation and hepatocellular carcinoma (HCC) development. However, their role in these processes is still unclear. In the present study, by using hepatocytes-specific DDB1 deletion mouse models, we explored the role and mechanism of IL6, TNFα and TNFR1 in oval cell proliferation and HCC development in the context of inflammation, which is the common features of HCC pathogenesis in humans. Our results showed that IL6 promotes oval cell proliferation and liver regeneration, while TNFα/TNFR1 does not affect this process. Deletion of IL6 accelerates HCC development and increases tumor burden. The number of natural killer(NK) cells is significantly decreased in tumors without IL6, implying that IL6 suppresses HCC by NK cells. In contrast to IL6, TNFR1-mediated signaling pathway promotes HCC development, and deletion of TNFR1 reduced tumor incidence. Increased apoptosis, compensatory proliferation and activation of MAPK/MEK/ERK cascade contribute to the oncogenic function of TNFR1-mediated signaling pathway. Intriguingly, deletion of TNFα accelerates tumor development, which shows divergent roles of TNFα and TNFR1 in hepatocarcinogenesis.

## INTRODUCTION

Cytokines modulate various cell types in the liver. Among them, interleukine-6 (IL6) and tumor necrosis factor α (TNFα), which are produced mainly by macrophage, may be the most critical ones [1]. They are implicated in both liver physiological and pathological processes, including regeneration and hepatocellular carcinoma (HCC) [2, 3].

Under liver pathological conditions, liver progenitor cells or oval cells, which have bi-potential capacity to differentiate into hepatocytes and cholangiocytes, are activated to restore liver mass [4]. TNFα and IL6 are suggested to promote oval cell proliferation in this process [5, 6]. However, due to incapable of distinguishing newborn hepatocytes from pre-exist ones, liver regeneration could not be fully assessed in these chemical injury models [7]. Furthermore, recent studies found that oval cells play minor if any contribution to liver regeneration in conventional chemicals injury models, including thionine-supplemented(CDE) and 3-diethoxycarbonyl-1,4-dihydrocollidine (DDC) [8]. Therefore, the roles of TNFα and IL6 in oval cells-mediated liver regeneration are required to be re-evaluated in an appropriate model, in which oval cells contribute a lot to liver regeneration.

In addition to liver regeneration, IL6 and TNFa/TNFR1 signaling pathways are also involved in HCC development [9]. Deletion of IL6 inhibited diethylnitrosamine(DEN)-induced HCC development in mouse [10]. TNFα and TNFR1 do not contribute to HCC development induced by DEN, but promote obesity-and long-term CDE-induced HCC development [11, 12]. Thus, IL6 and TNFα/TNFR1 signaling pathways promote HCC development in most mouse models. However, HCC patients with high level of IL6 and TNFα have better prognosis [13–15]. 90% of HCC cases in human arised in the context of hepatic inflammation [16], while in chemicals induced HCC models, tumors arise from chemically induced mutation without initial underlying hepatitis [17]. Therefore, re-evaluating the role of IL6, TNFα and TNFR1 in mouse model whose HCC development is accompanied with inflammation is more appropriately.

To re-evaluate the role of cytokines in oval cells-mediated liver regeneration and HCC development in inflammatory condition, we explored IL6 and TNFα/TNFR1 signaling in genetic induced hepatocyte damage model. Hepatocyte-specific deletion of damaged-binding protein 1(DDB1), an adaptor protein for Cullin4 ubiquitin ligase, blocks the proliferation ability of hepatocytes, resulting in compensatory regeneration mediated by oval cells and progressive tumor development in the context of inflammation [18]. Newborn hepatocytes derived from oval cells could be genetically distinguished in our model, and oval cells were induced independent of chemicals excluding the promiscuous effects of chemicals [7]. In addition, persistent deletion of DDB1 in *DDB1^F/F^, Alb-Cre^+/−^* mouse results in HCC arises in aged mice, preceding by intra-hepatic inflammation and immune cells infiltration [18]. Herein, by using hepatocyte-specific DDB1 knockout models, we reported divergent roles of IL6, TNFα and TNFR1 in oval cells-mediated liver regeneration and inflammation-associated hepatocarcinogenesis.

## RESULTS

### IL6 deficiency delayed liver generation in *DDB1^F/F^, Mx1-Cre^+/−^* Mice after poly(I:C) injection

**Figure 1:**
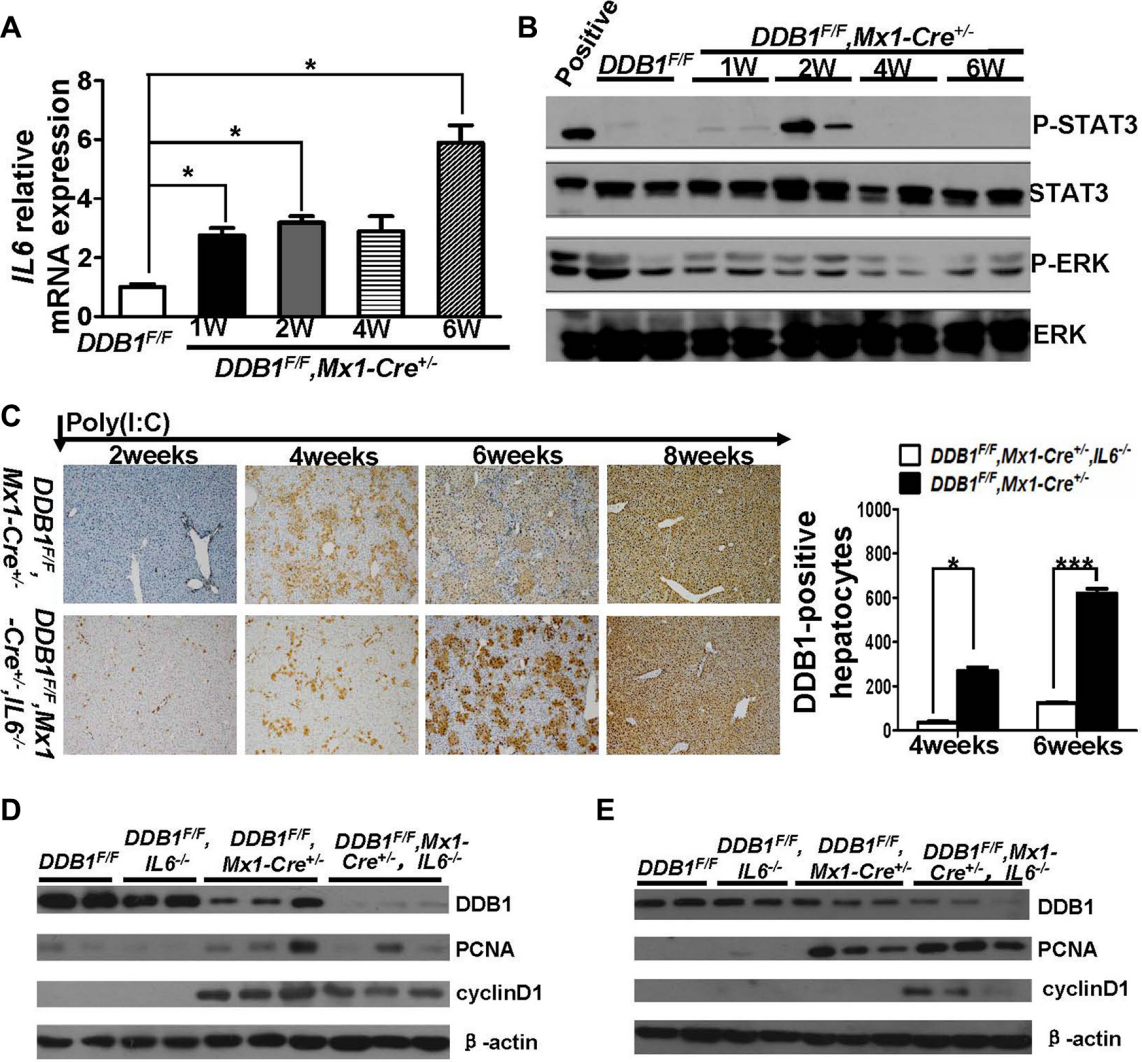
Deletion of IL6 delayed liver regeneration in DDB1^F/F^, Mx1-Cre^*+/−*^ mouse after poly(I:C) injection (**A**) The mRNA level of IL6 at indicated time points after poly(I:C) injection was detected by RT-PCR. Results are represented as mean ± S.E.M, *n* = 3–6, *P < .0.05. (**B**) The activation of IL6 downstream proteins STAT3 and ERK were detected by western blot. (**C**) IHC staining for DDB1 in liver slides(magnification,100×) and DDB1-positive hepatocytes counting at indicated time points after poly(I:C) injection, cells were counted under 20× objective from eight independently visual fields. Data are represented as mean ± S.E.M, *n* = 3–6, *P < 0.05, ***P < 0.001. Western blot for detecting DDB1, PCNA and cyclinD1 in liver homogenates of indicated mice at 4 weeks (**D**) and 6 weeks (**E**) after poly(I:C) injection.

We previously reported that injection of poly(I:C) into *DDB1*^F/F^, *Mx1-Cre^+/−^* mouse induced hepatocyte-specific DDB1 deletion. Oval cells are activated and differentiated into DDB1 positive hepatocytes subsequently [18]. Expression of IL6 was upregulated in the liver of *DDB1^F/F^, Mx1-Cre^+/−^* mice after poly(I:C) injection, with activation of downstream STAT3 but not ERK signaling (Figure 1A, 1B). To investigate the role of IL6 in oval cells mediated liver regeneration, *DDB1^F/F^, Mx1-Cre^+/−^, IL6^−/−^* mouse was obtained. DDB1 positive hepatocytes depletion was achieved in both IL6 normal and deficient *DDB1^F/F^, Mx1-Cre^+/−^* mice two weeks after poly(I:C) injection (Figure 1C). Newborn DDB1-positive hepatocytes were observed with much fewer in *DDB1^F/F^, Mx1-Cre^+/−^, IL6^−/−^* mice at 4 and 6 weeks post injection (Figure 1C). The level of DDB1 positive hepatocytes in IL6 deficient mice was regenerated to the same level as IL6 normal mice until 8 weeks post injection (Figure 1C). The delayed regeneration is due to slow proliferation as lower level of proliferation markers PCNA and cyclinD1 in *DDB1^F/F^, Mx1-Cre^+/−^, IL6^−/−^* mice at 4 weeks post injection, which was recovered in IL6 deficient mice at 6 weeks post injection (Figure 1D, 1E). Taken together, these data indicated that IL6 is required for liver regeneration in *DDB1^F/F^, Mx1-Cre^+/−^* mouse, loss of IL6 would delay this process.

### Oval cell proliferation was inhibited in *DDB1^F/F^, Mx1-Cre^+/−^, IL6*^−/−^ *mouse*

**Figure 2:**
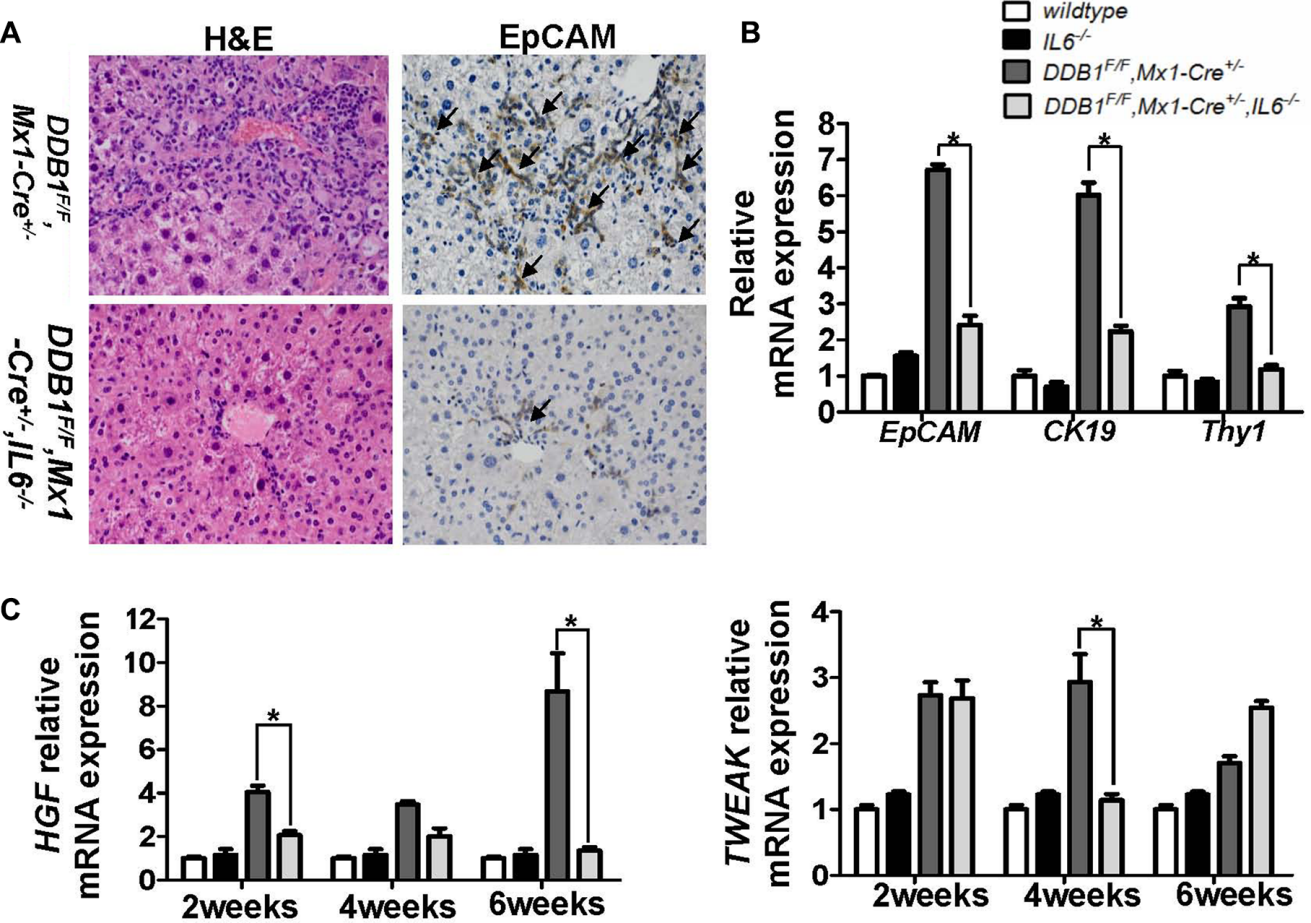
IL6 promotes oval cell proliferation by elevating the expression of HGF and TWEAK (**A**) Liver tissues of indicated mice at 6 weeks after poly(I:C) injection were stained with HE and EpCAM(magnificantion, 400×, arrows indicated positive cells), and (**B**) the mRNA levels of EpCAM, CK19 and Thy1 were quantified by RT-PCR. Data are represented as mean ± S.E.M, *n* = 3–6, *P < 0.05. (**C**) The hepatic mRNA levels of HGF and TWEAK at indicated time points after poly(I:C) injection were measured by RT-PCR. Results are represented as mean ± S.E.M, *n* = 3–6, *P < 0.05.

Newborn hepatocytes are mainly derived from oval cells in *DDB1^F/F^, Mx1-Cre^+/−^* mouse after poly(I:C) injection. To determine whether delayed liver regeneration in *DDB1^F/F^, Mx1-Cre^+/−^, IL6^−/−^* mouse is due to restricted oval cell proliferation, EpCAM expression, a biomarker of oval cells, was measured. Compared to *DDB1^F/F^, Mx1-Cre^+/−^* mouse, EpCAM^+^ oval cells were diminished in *DDB1^F/F^, Mx1-Cre^+/−^, IL6^−/−^* mouse (Figure 2A). Restricted proliferation of oval cells was further confirmed by significant reduction of CK19 and Thy1 (Figure 2B). Furthermore, upregulation of HGF and TWEAK, two important factors for oval cell proliferation, was significantly attenuated by IL6 deletion(Figure 2C). Collectively, these results suggested that IL6 is required for oval cell proliferation, partly by promoting the expression of HGF and TWEAK.

### TNFR1 is dispensable for oval cell proliferation and liver regeneration in *DDB1^F/F^, Mx1-Cre^+/−^* mouse

**Figure 3:**
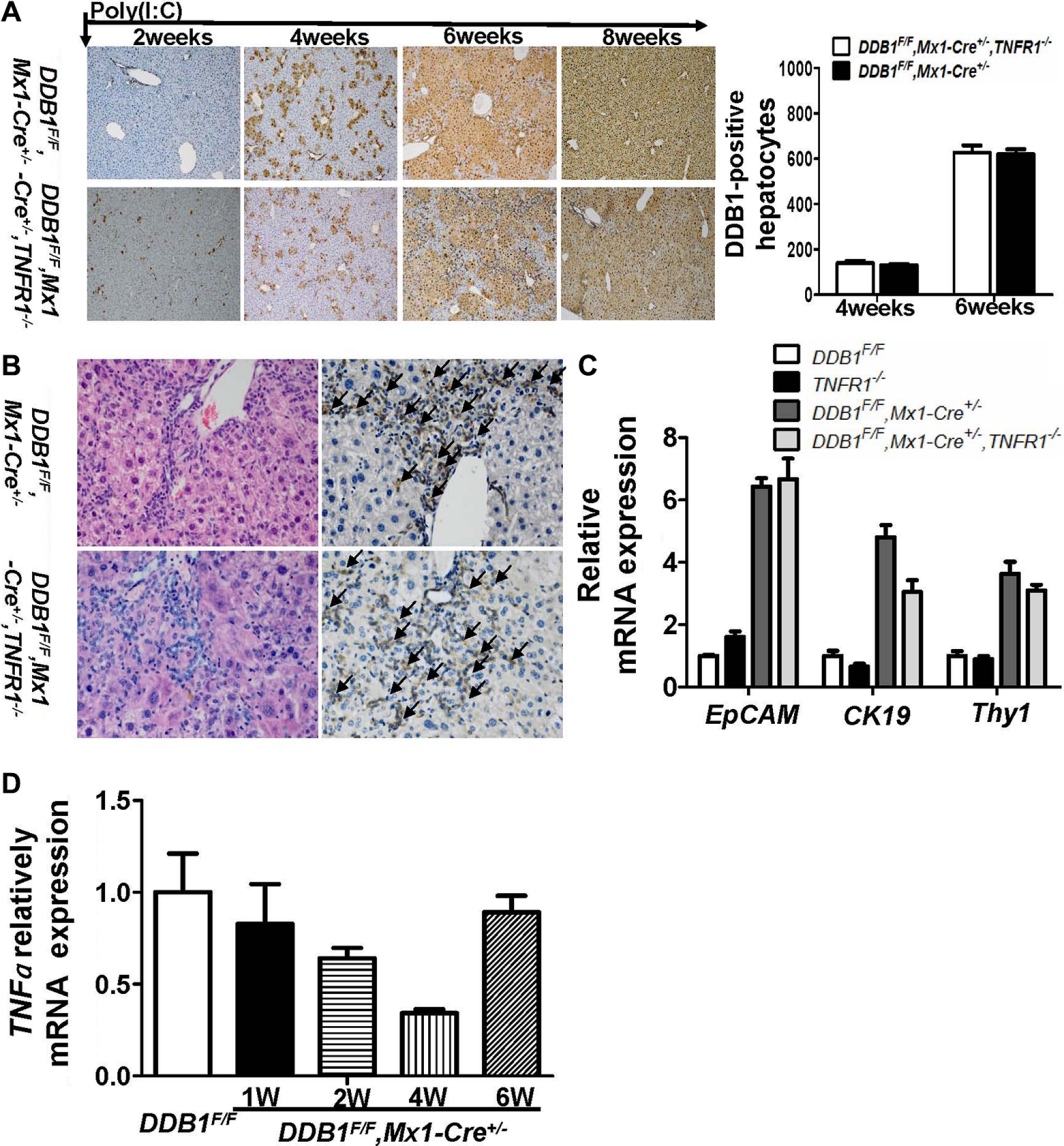
TNFR1 was dispensable for oval cell proliferation in *DDB1^F/F^, Mx1-Cre^+/−^* mouse (**A**) IHC staining for DDB1 in liver tissues at indicated time points after poly(I:C) injection(magnification,100×) and DDB1-positive hepatocytes counting, cells were counted under 20× objective from eight independently visual fields. Data are represented as mean ± S.E.M, *n* = 4. (**B**) HE and IHC staining for EpCAM in liver tissues at 6 weeks after poly(I:C) injection (magnificantion, 400×, arrows indicated positive cells). (**C**) The mRNA levels of EpCAM, CK19 and Thy1 were quantified by RT-PCR. Data are represented as mean ± S.E.M, *n* = 4. (**D**) Detecting mRNA level of TNFα at indicated time points after poly(I:C) injection by real time RT-PCR. Results are represented as mean ± S.E.M, *n* = 4, *P < .0.05.

The role of TNFR1 in oval cell proliferation and liver regeneration was investigated as IL6. Equal DDB1-positive hepatocytes were regenerated at various time points after poly(I:C) injection with similar oval cell distribution in both TNFR1 wildtype or deficient DDB1^*F/F*^, Mx1-Cre^*+/−*^ mice (Figure 3A–3C). The expression of TNFα was also not varied significantly after poly(I:C) injection, as shown in Figure 3D. These results suggested that TNFα/TNFR1-mediated signaling pathway was dispensable for oval cell proliferation and liver regeneration in *DDB1^F/F^, Mx1-Cre^+/−^* mouse.

### Intra-hepatic inflammation and immune cells infiltration before tumors arise in *DDB1^F/F^, Alb-Cre^+/−^* mouse

**Figure 4:**
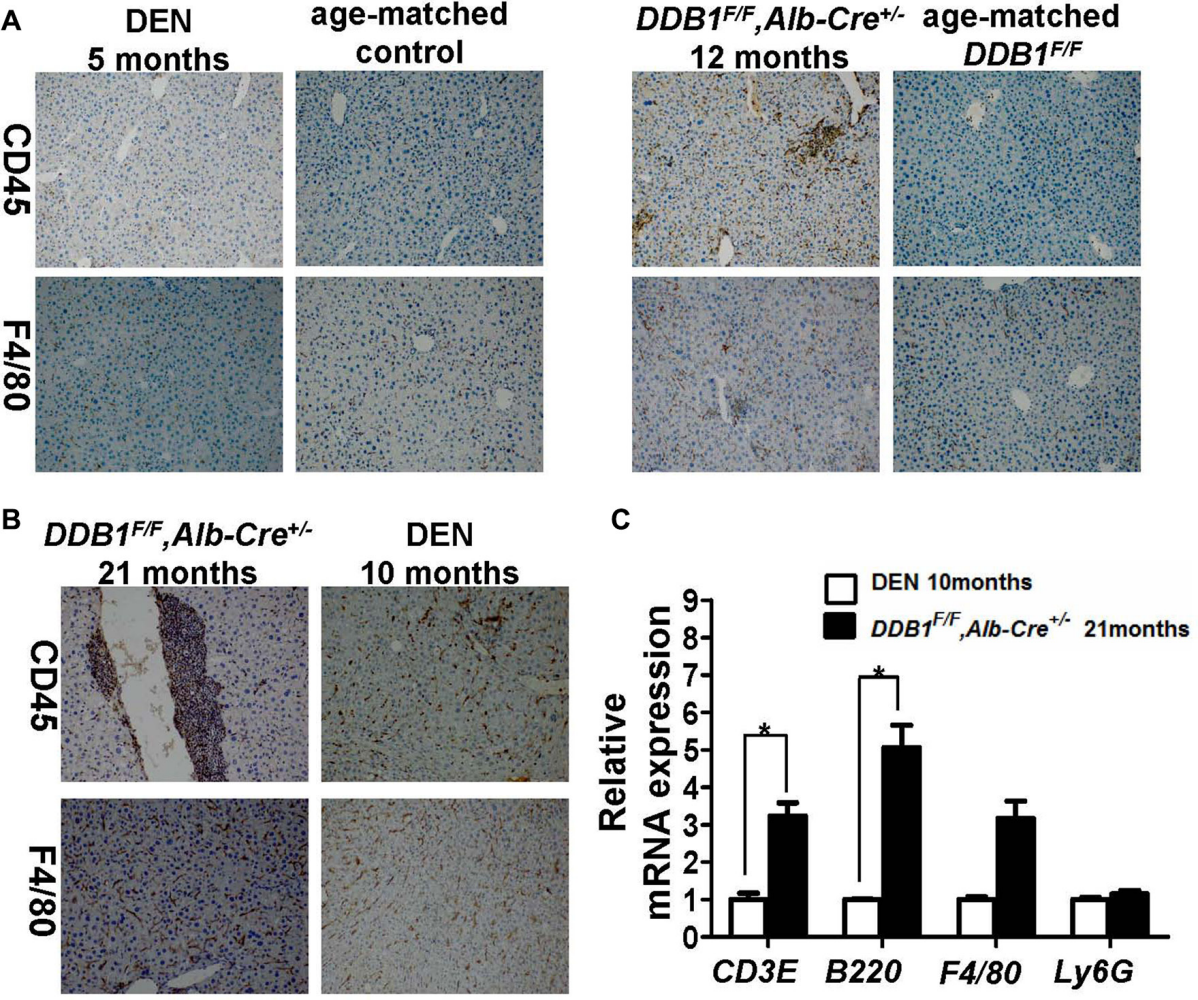
Established inflammation before HCC arises and more immune cells infiltration in *DDB1^F/F^, Alb-Cre^+/−^* mouse (**A** and **B**) Representative pictures of IHC staining for CD45 and F4/80 in liver slides of indicated mice (magnification, 100×). (**C**) The hepatic mRNA levels of CD3E, B220, F4/80 and Ly6G were measured by RT-PCR. Data are represented as mean ± S.E.M, *n* = 4, *P < 0.05.

Various types of cancer arise under context of inflammation, especially for HCC [19]. Inflammation before tumor arised in *DDB1^F/F^, Alb-Cre^+/−^* and DEN mouse model was assessed, as shown in Figure 4A, by IHC for biomarkers of leukocytes(CD45) and macrophage (F4/80), more inflammatory cells in 12 months old *DDB1^F/F^, Alb-Cre^+/−^* mouse than age-matched control were observed, while no difference of inflammatory cells between DEN-treated and age-match control. We also evaluated the level of inflammation when noticeable HCC developed by IHC and RT-PCR. As shown Figure 4B and 4C, more inflammatory cells infiltration and T/B cells were detected in *DDB1^F/F^, Alb-Cre^+/−^* mouse. These data suggested that compared to DEN model, *DDB1^F/F^, Alb-Cre^+/−^* mouse model recapitulates key features of human HCC pathogenesis in inflammation.

### IL6 suppresses HCC in *DDB1^F/F^, Alb-Cre^+/−^* mouse through NK cells-mediated tumor surveillance

**Figure 5:**
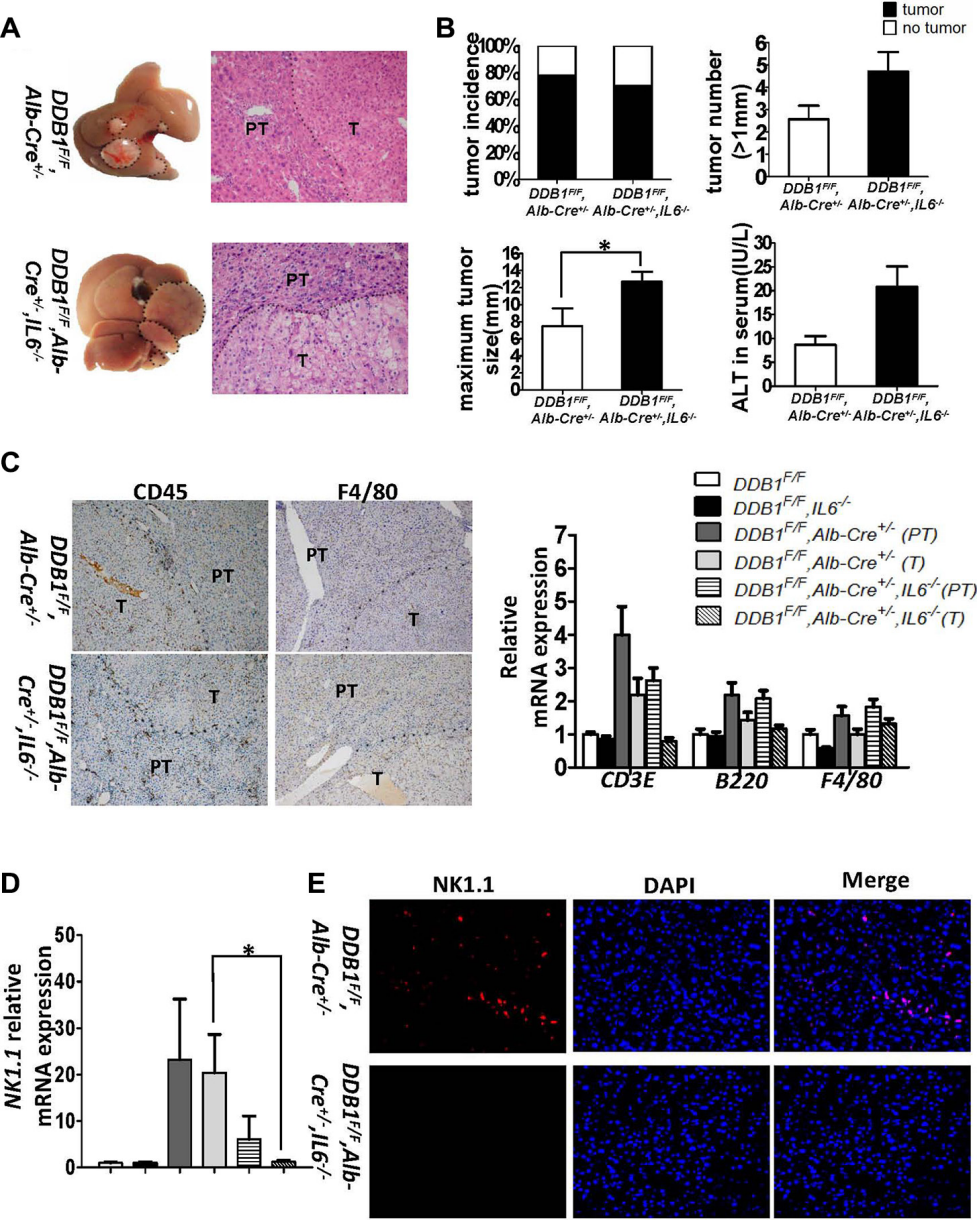
IL6 suppressed inflammation-associated HCC development by modulating NK cells (**A**) Representative pictures of liver appearance and HE staining of liver slides(magnification, 400×).(**B**) Tumor incidence, tumor number(> 1 mm), maximum tumor size and ALT in serum of indicated mice aged 21 months, dotted lines indicated tumor. Data are represented as mean ± S.E.M, *n* = 4–5, *P < 0.05. (**C**) Representative pictures of IHC for CD45 and F4/80 (magnification, 100×) and the mRNA levels of CD3E, B220, F4/80 detected by RT-PCR T indicates tumor and PT indicates para-tumor. Data are represented as mean ± S.E.M, *n* = 4–5. (**D**) The mRNA level of NK1.1 was detected by RT-PCR. Data are represented as mean ± S.E.M, *n* = 4–5, *P < 0.05. (**E**) IF staining for NK1.1 in tumor tissues of indicated mice. Representative pictures are shown (magnification, 200×).

**Figure 6:**
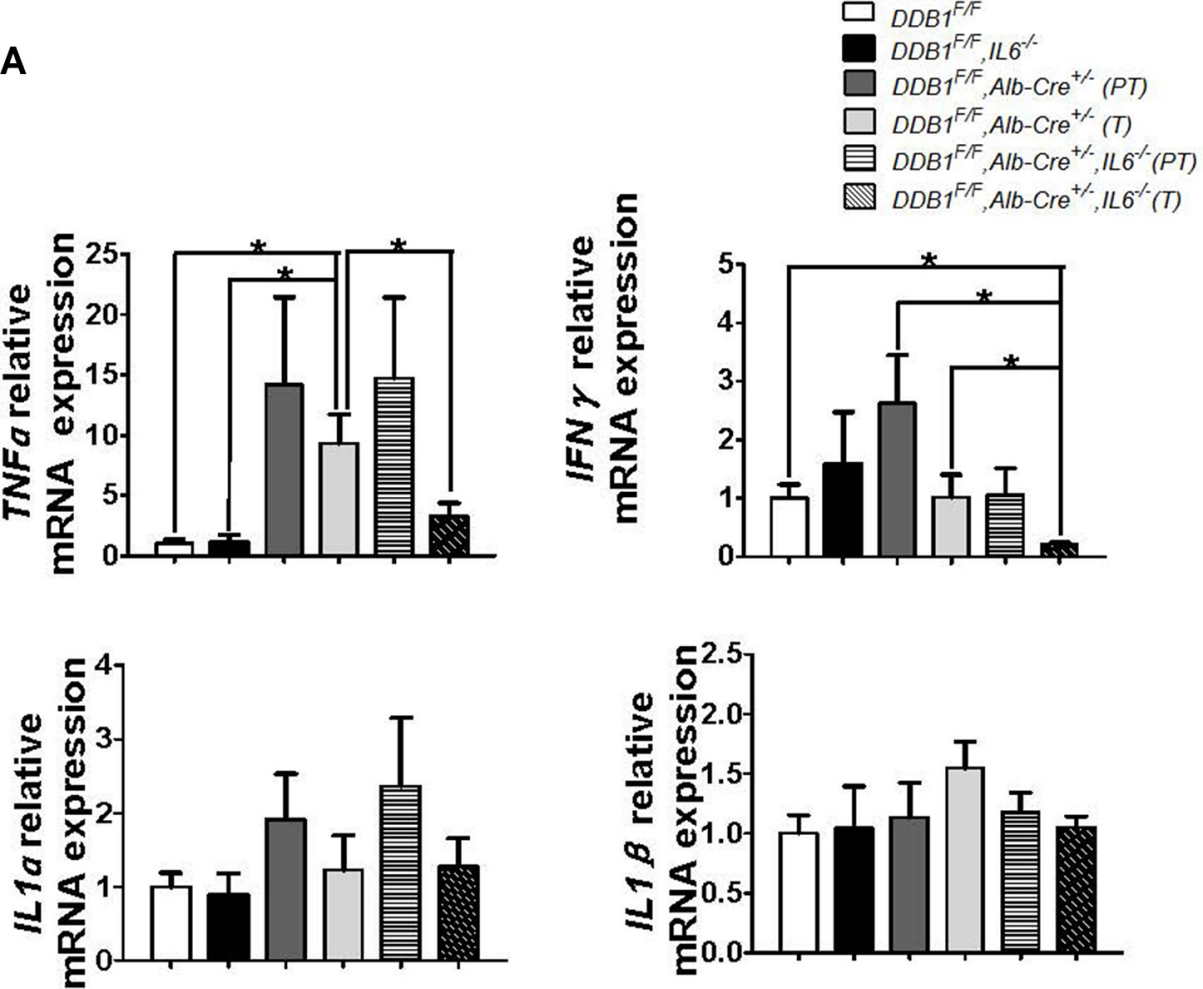
The expression of TNFα and IFNγ was significantly reduced in tumor regions of *DDB1^F/F^, Alb-Cre^+/−^, IL6^−/−^* mouse (**A**) The mRNA levels of TNFα, IFNγ, IL1α and IL1β of liver tissues of indicated mice were detected by RT-PCR. Data are represented as mean ± S.E.M, *n* = 4–5, *P < 0.05.

*DDB1^F/F^, Alb-Cre^+/−^, IL6^−/−^* mouse was obtained to investigate the role of IL6 in inflammation-associated HCC development. More *DDB1^F/F^, Alb-Cre^+/−^, IL6^−/−^* mice developed HCC at the age of 18 months (Supplementary Figure S1). At age of 21 months, even the tumor incidence was similar, the maximum tumor size increased significantly in *DDB1^F/F^, Alb-Cre^+/−^, IL6^−/−^* mouse (Figure 5A, 5B). Although Liver injury was elevated by deletion of IL6 reflected in ALT measurement, the apoptosis and compensatory proliferation was unaltered with similar level of cleaved-caspase3 and PCNA in IL6 wildtype and deficient *DDB1^F/F^*,*Alb-Cre^+/−^* mice (Supplementary Figure S2). Inflammation was also measured by IHC with similar staining for CD45 and F4/80 (Figure 5C). Interestingly, NK cells infiltration was decreased significantly in tumors of *DDB1^F/F^, Alb-Cre^+/−^, IL6^−/−^* mouse compared to *DDB1^F/F^, Alb-Cre^+/−^* mouse, confirmed by RT-PCR and IF for NK1.1, the biomarker of NK cells (Figure 5D, 5E). Finally, intra tumor TNFα and IFNγ level was significantly reduced in *DDB1^F/F^, Alb-Cre^+/−^, IL6^−/−^* mouse comparing to *DDB1^F/F^, Alb-Cre^+/−^* mouse (Figure 6). Taken together, our results suggested that deletion of IL6 promoted HCC development and increased tumor burden, NK cells, TNFα and IFNγ were significantly reduced in tumors of *DDB1^F/F^, Alb-Cre^+/−^, IL6^−/−^* mouse.

### TNFR1 and TNFα show divergent role in HCC development in *DDB1^F/F^, Alb-Cre*^*+/−*^ mouse

**Figure 7:**
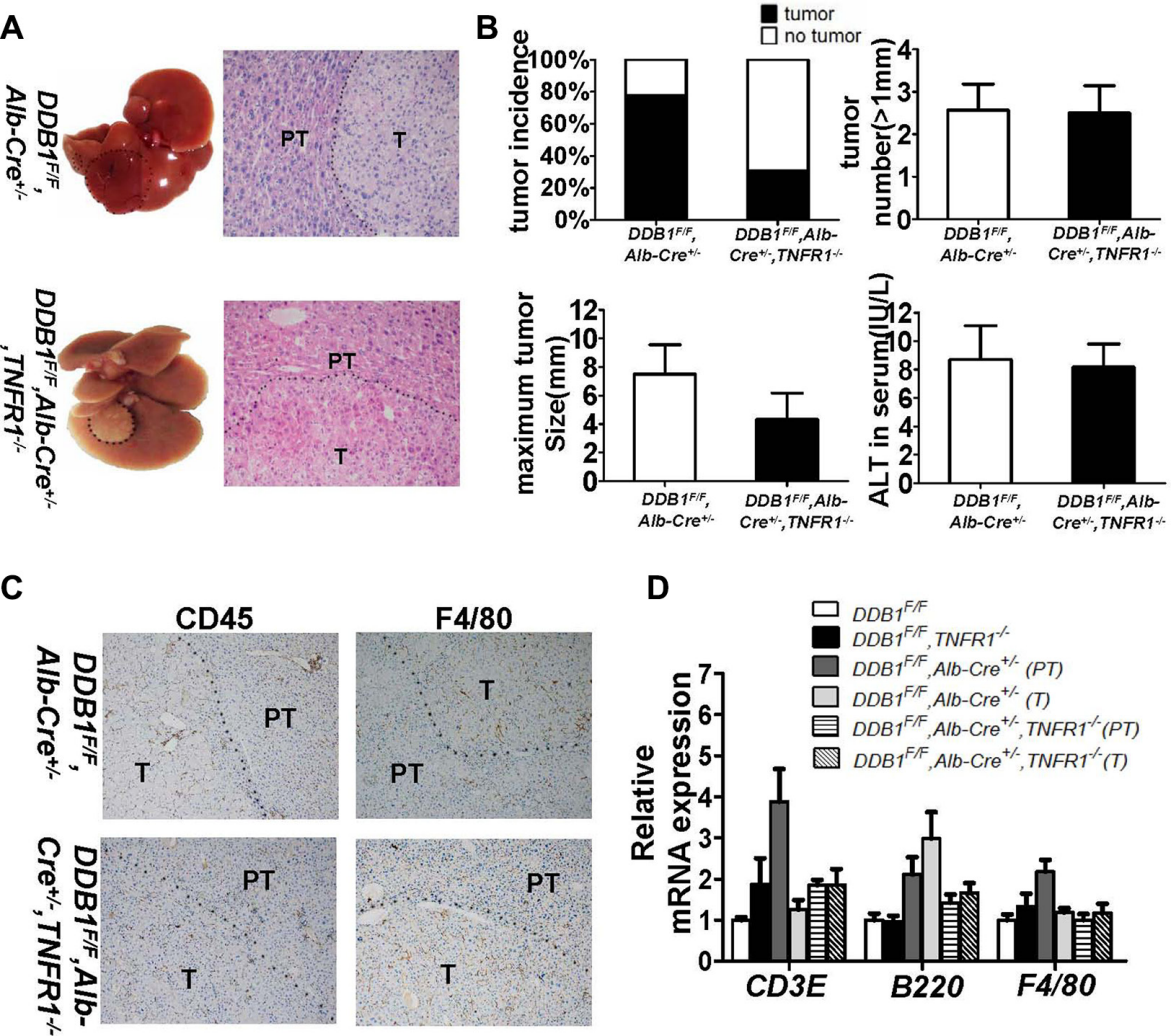
Deletion of TNFR1 reduced HCC incidence in *DDB1^F/F^, Alb-Cre*^+/−^ mouse All mice were sacrificed at the age of 21 months, tumor incidence, tumor numbers (> 1 mm) and maximum tumor size were calculated. (**A**) Representative pictures of liver and HE staining of liver slides (magnification, 400×), dotted line indicating tumor. (**B**) Tumor incidence, tumor number (> 1 mm), maximum tumor size and ALT in serum of indicated mice at the age of 21 months. Data are represented as mean ± S.E.M, *n* = 3–4. (**C**) Representative pictures of IHC for CD45 and F4/80 (magnification, 100×) and (**D**) the mRNA levels of CD3E, B220, F4/80 detected by RT-PCR, T indicates tumor and PT indicates para-tumor. Data are represented as mean ± S.E.M, *n* = 3–4.

**Figure 8:**
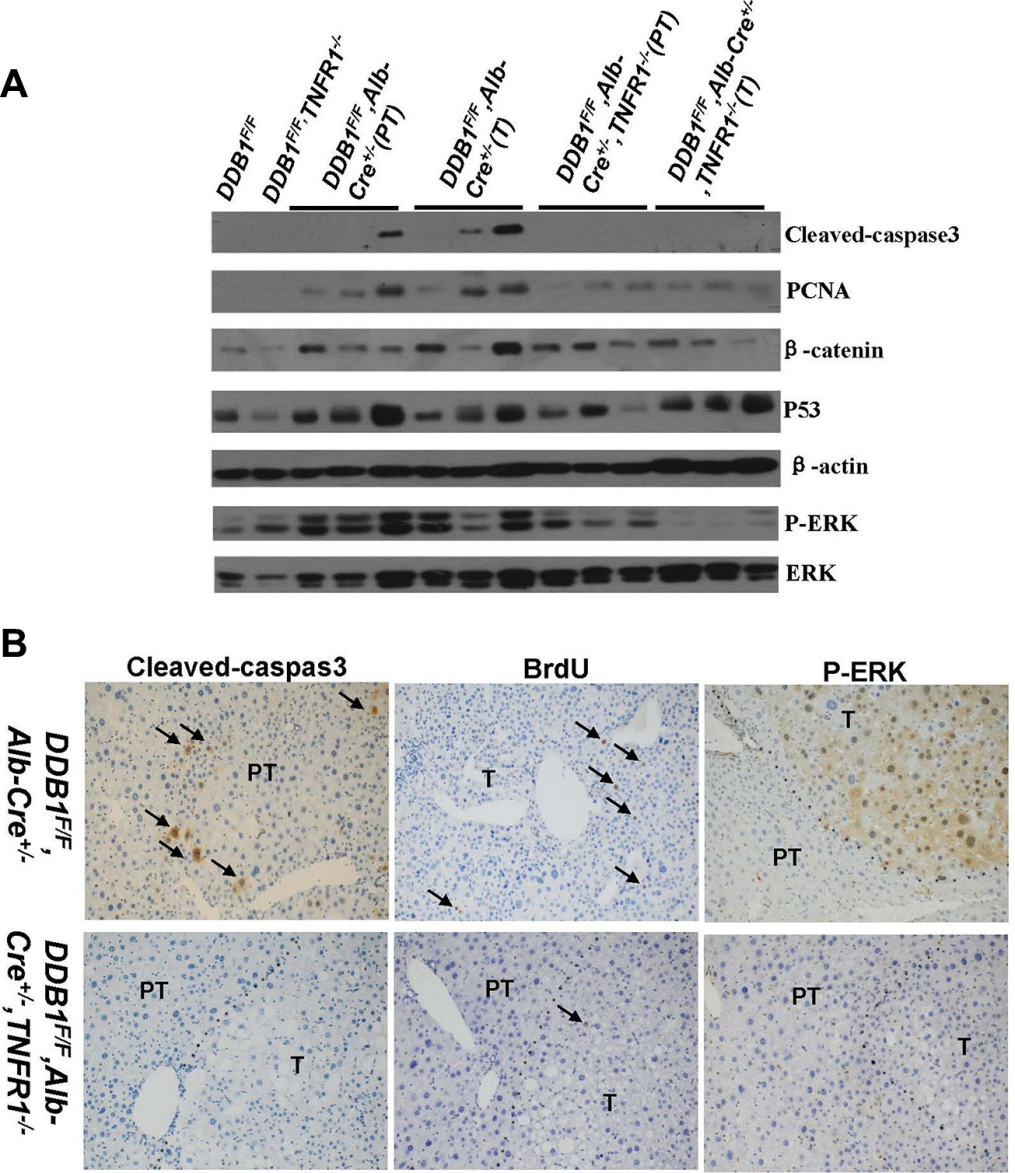
Reduction of cell apoptosis and compensatory proliferation, P-ERK level in DDB1^F/F^, Alb-Cre^+/−^, TNFR1^−/−^ mouse (**A**) Western blot for detecting cleaved-caspase3, PCNA, β-catenin, P-ERK and ERK. (**B**) Representative pictures of IHC for cleaved-caspase3, BrdU and P-ERK (magnification, 400×), arrows indicate positive cells

**Figure 9:**
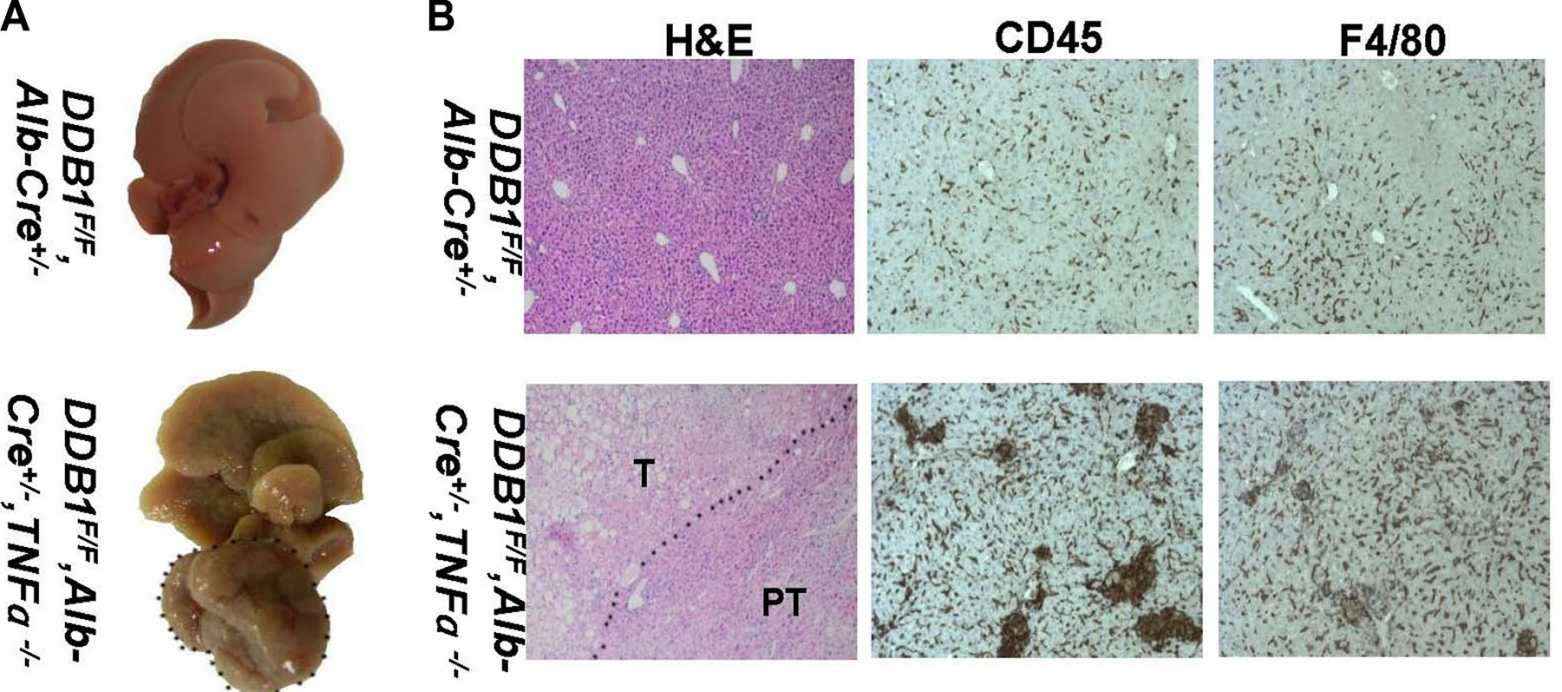
Deletion of TNFα accelerated tumor development in *DDB1^F/F^, Alb-Cre*^+/−^ mouse (**A**) Representative pictures of liver, dotted line indicates tumor. (**B**) Representative pictures of HE staining and IHC for CD45 and F4/80 (magnification, 200×). T indicates tumor and PT indicates para-tumor.

We investigated the role of TNFR1 in inflammation-associated HCC development in *DDB1^F/F^, Alb-Cre^+/−^,* TNFR1^*−/−*^ mouse. TNFR1 knockout reduced tumor incidence significantly, from 78% in *DDB1^F/F^, Alb-Cre^+/−^* mouse to 31% in *DDB1^F/F^, Alb-Cre^+/−^, TNFR1^−/−^* mouse (Figure 7A, 7B). NK cell infiltration was not affected by TNFR1 deletion (Supplementary Figure S3). Inflammation was similar between *DDB1^F/F^, Alb-Cre*^+/−^*, TNFR1^−/−^* and *DDB1^F/F^, Alb-Cre^+/−^* mouse (Figure 7C, 7D), with the same Th1 cytokine expression detected by RT-PCR (Supplementary Figure S4). However, cell apoptosis and compensatory proliferation was significantly decreased in *DDB1^F/F^, Alb-Cre^+/−^, TNFR1^−/−^* mouse with reduced cleaved-caspase3, BrdU and PCNA (Figure 8A, 8B). Furthermore, P-ERK was also reduced significantly in *DDB1^F/F^, Alb-Cre^+/−^, TNFR1^−/−^* mouse (Figure 8A, 8B). In contrast to TNFR1 knockout, deletion of TNFα accelerated HCC development in *DDB1^F/F^, Alb-Cre^+/−^* mouse. 13 months old *DDB1^F/F^, Alb-Cre^+/−^, TNFα^−/−^* mice developed tumors with massive inflammatory cell infiltration, while normal morphology in age-matched *DDB1^F/F^, Alb-Cre^+/−^* mice (Figure 9). Taken together, our results suggested that TNFR1 elevated HCC incidence, partially by promoting cells turnover and upregulating P-ERK. Unlike TNFR1, deletion of TNFα accelerated HCC development, which showed divergent role of TNFα and TNFR1 in hepatocarcinogenesis.

## DISCUSSION

The involvement of IL6, TNFα and TNFR1 in oval cells mediated liver regeneration and HCC development has been explored by numerous studies with controversial results and discrepancy between experimental and clinical data [13, 20, 21]. By using hepatocyte-specific DDB1 deletion mouse models, we found IL6 promotes oval cells-mediated liver regeneration, while TNFα/TNFR1 signaling pathway does not affect this process. Intriguingly, TNFα and TNFR1showed divergent role in inflammation-associated hepatocarcinogenesis, and for the first time, the anti-tumor function of IL6 in HCC was revealed in present study.

Recently, Lu et al. reported that deletion of Mdm2, an E3 ubiquitin ligase responsible for P53 degradation, in hepatocytes resulted in P53 accumulation and elicited cell death and senescence. Subsequently, oval cells were induced and differentiated into P53 negative hepatocytes to restore liver mass [22]. Similarly, hepatocyte-specific deletion of DDB1 also induced cell senescence(unpublished data), followed by oval cells-mediated liver regeneration. The regenerative process in *DDB1^F/F^, Mx1-Cre^+/−^* mouse is characterized by replenishment of DDB1 negative hepatocytes with positive ones. Distinct from chemicals injury models, oval cells are the major source of newborn hepatocytes, and liver regeneration could be genetically assessed [7, 18]. Therefore, *DDB1^F/F^, Mx1-Cre^+/−^* mouse model suits the investigation for oval cells mediated liver regeneration.

During hepatocytes reconstitution, elevated expression of IL6 was detected, which may secreted by inflammatory cells or senescent hepatocytes [23]. IL6 is shown to promote liver regeneration after partial hepatectomy [24] and oval cell proliferation induced by CDE diet [5]. Therefore, we speculated that IL6 is required for oval cells-mediated liver regeneration in *DDB1^F/F^, Mx1-Cre^+/−^* mouse. This hypothesis was confirmed by IL6 depletion, which showed delayed liver regeneration and restricted oval cell proliferation. Previous studies have suggested IL6 promotes oval cell proliferation by activating JAK/STAT3 signaling [5]. Consistently, activation of JAK/STAT3 was observed in *DDB1^F/F^, Mx1-Cre^+/−^* mouse at 1 and 2 weeks post poly(I:C) injection. In addition, expression of HGF and TWEAK, two important mitogens for oval cells, was attenuated by IL6 depletion. HGF and TWEAK are secreted by stellate cells and macrophages respectively, their contribution on oval cell proliferation has been well characterized [25, 26]. Therefore, IL6 also modulate oval cell proliferation by promoting HGF and TWEAK expression *in vivo*. Unlike IL6, TNFR1 depletion did not affect liver regeneration in *DDB1^F/F^, Mx1-Cre^+/−^* mouse. The expression of TNFα was also unaltered during regeneration. This results indicated that TNFα/TNFR1 signaling pathway is dispensable for oval cells-mediated liver regeneration in *DDB1^F/F^, Mx1-Cre^+/−^* mouse.

Although IL6 promotes DEN-induced HCC development, its anti-tumor role in hepatocarcinogenesis was observed in current study. IL6 depletion accelerated tumor development and increased tumor burden in *DDB1^F/F^, Alb-Cre^+/−^* mouse. Detailed analysis found liver injury, compensatory proliferation, and intra-hepatic inflammation was not aggravated in *DDB1^F/F^, Alb-Cre^+/−^, IL6^−/−^* mouse. Interestingly, NK cells were decreased significantly in tumors without IL6. NK cells play an important role in tumor surveillance, it exerts anti-tumor function by direct cytotoxicity or cytokine secretion such as IFNγ [27]. In HCC patients, high density of intra-tumoral NK cells correlated with long survival rate [28]. To date, the relationship between NK cells and IL6 is still unclear and controversial [29–31]. Based on our results, we postulated that IL6 signaling modulates NK cells to inhibiting inflammation-associated hepatocarcinogenesis, the underlying mechanism will be our next step.

Differ from IL6, TNFR1 depletion reduced HCC incidence in *DDB1^F/F^, Alb-Cre^+/−^* mouse, accompanied with decreased cell death/compensatory proliferation and inhibited activation of MAPK/MEK/ERK cascade. The oncogenic function of TNFR1 may mediate through cell death/compensatory proliferation and MAPK/MEK/ERK cascade, due to the positive correlation between them and hepatocarcinogenesis [32–34]. Although TNFR1 signaling is mainly initiated by TNFα, which is suggested to promote HCC development in *mdr2^−/−^* mouse [35], deletion of TNFα accelerated rather than inhibited hepatocarcinogenesis in *DDB1^F/F^, Alb-Cre^+/−^* mouse. The divergent role of TNFα and TNFR1 implies that other ligands bind TNFR1 to promote HCC development, probably lymphotoxin-α, whose overexpression with lymphotoxin-β elicited HCC spontaneously [36].

Taken together, by using hepatocyte-specific DDB1 deletion mouse models, the roles of IL6 and TNFα/TNFR1 signaling in liver regeneration and hepatocarcinogenesis were systematically investigated. IL6 promotes liver regeneration by stimulating oval cell proliferation, while TNFα/TNFR1 signaling is dispensable for this process. For the first time, the anti-tumor role of IL6 in hepatocarcinogenesis was suggested, and NK cells were shown to involve in. Although TNFR1 promotes HCC development in *DDB1^F/F^, Alb-Cre^+/−^* mouse, TNFα depletion accelerates rather than inhibits hepatocarcinogenesis. Other ligands may initiate oncogenic function of TNFR1 signaling and special cautions should be paid to anti-IL6 and anti-TNFα therapy in HCC patients.

## MATERIALS AND METHODS

### Mice

*DDB1^F/F^, Alb-Cre^+/^*^−^ and *Mx1-Cre^+/−^* mice were constructed previously [18]. *IL6^−/−^*, *TNFα^−/−^* and *TNFR1^−/−^* mice are acquired from Jackson Laboratory. I*L6*^−/−^ *, TNFα^−/−^* and *TNFR1^−/−^* mice were crossed with *DDB1^F/F^, Alb-Cre*^+/−^ and *Mx1-Cre^+/−^* mice respectively to obtain double mutant mice. All animals were maintained in pathogen-free facilities and all experiments were conducted according to the Guide for the Care and Use of Laboratory Animals prepared by the National Academy of Sciences and published by NIH (publication 86–23 revised 1985).

### Experimental protocol

For deletion of DDB1 in *DDB1^F/F,^Mx1-Cre^+/−^* and *DDB1^F/F,^ Mx1-Cre^+/−,^TNFR1^−/−^ &IL6^−/−^* mice, 6–8 weeks mice were i.p. injected polyinosinic:polycytidylic acid (poly(I:C) (GE healthcare) at 13 mg/kg mouse weight. For DEN model, DEN (sigma) was injected intraperitoneally into 2 weeks old male mice at a dose of 25 mg/kg mouse weight. For BrdU incorporation assays, mice were injected intraperitoneally with 100 mg/kg BrdU and sacrificed 1 h later.

### Western blot analysis

Liver tissues were homogenized in RIPA buffer (sigma) containing protease inhibitor cocktail (Roche), incubated on ice for 30 min, and centrifuged for 15 min at 12,000 g, 4°C. Protein concentration was quantified by BCA protein kit (Thermo). Equal amounts of protein were separated discontinuously on 4–12% SDS–PAGE gel and transferred to polyvinylidene difluoride membrane (Millipore). The membranes were incubated overnight at 4°C with the desired primary antibodies, washed three times with TBS/T and incubated with the appropriate HRP-conjugated secondary antibodies (Santa Cruz) for 1 hour at room temperature. Protein level was detected by chemiluminescence (Pierce Biotechnology). The antibodies used for Western blotting include: anti-p44/42 MAPK (Erk1/2) (Thr202/Tyr204), anti-p44/42 MAPK (Erk1/2), anti-β-catenin, anti-P-STAT3(Thr705) and anti-STAT3(Cell Signaling Technology), anti-PCNA, anti-p53(Santa Cruz), anti-DDB1 and anti-β-actin(Abcam).

### Immunohistochemistry and immunofluorescence

Five-micrometer paraffin tissue sections were prepared. After blocking endogenous peroxidase activity and non-specific staining, the sections were incubated overnight at 4°C with the primary antibodies. Immunohistochemistry (IHC) was done using the streptavidin-biotin peroxidase complex method according to manufacturer's instructions (Lab Vision). For immunofluorescence (IF), slides were incubated with DAPI for 3 minutes after incubating with fluorescein-conjugated primary antibodies, washed with PBS and then covered with anti-fade(Sangon Biotech). Antibodies used for immunohistochemistry were: anti-CD45 and anti-F4/80(eBioscience), anti-EpCAM (Abcam), anti-p44/42 MAPK (Erk1/2) (Thr202/Tyr204) (Cell Signaling Technology), biotin conjugated anti-BrdU (Millipore), anti-cleaved-caspase3 (Cell signaling Technology) and anti-DDB1 (Bethyl Laboratories). PE-Cy7 conjugated anti-NK1.1 (eBioscience) was used for immunofluorescence.

### Real Time-PCR (RT-PCR)

Total RNA was purified from liver tissue samples using Trizol (Invitrogen) according to manufacturer's protocol. Reverse transcript was performed using PrimeScript RT Master Mix kit (Takara). Real time PCR was carried out in SYBR Green PCR Master Mix (Bio-Rad) with ABI PRISM 7500 Sequence Detection System. Primer sequences are listed in Supplementary Table S1. Each measurement was performed in triplicate and results were normalized to the expression of *gapdh* reference gene.

### Serum alanine transaminases assay

Blood collected from mice incubated in room temperature for 2 hours, then centrifuge at 2000 g for 20 minutes. Serum in the upper layer after centrifuge transferred to new tubes. The activity of alanine transaminase (ALT) in serum was measured by alanine transaminases assay kit (Nanjin jiancheng).

### Statistical analysis

Data were analyzed using SPSS and represented as the mean ± SEM. Comparisons between two groups were performed using an unpaired Student's *t*-test. *P* < 0.05 was considered statistically significant

## SUPPLEMENTARY MATERIAL TABLE AND FIGURES


