# Supplementary material for: Distinct role of interleukin-6 and tumor necrosis factor receptor-1 in oval cell- mediated liver regeneration and inflammation-associated hepatocarcinogenesis
Source: Oncotarget. 2016 Aug 18;7(41):66635–46. doi: 10.18632/oncotarget.11365 (PMC5341826; doi:10.18632/oncotarget.11365)
Supplement: Supplementary file 1 [file oncotarget-07-66635-s001.pdf]

Distinct role of interleukin-6 and tumor necrosis factor receptor-1 in oval cell- mediated liver regeneration and inflammation-associated hepatocarcinogenesis

Supplementary Materials

Supplementary Table S1: RT-PCR primer pairs

Gene	Forward(5'-3')	Reverse(5'-3')
GAPDH	AGGTCGGTGTGAACGGATTTG	TGTAGACCATGTAGTTGAGGTCA
IL6	TCTATACCACTTCACAAGTCGGA	GAATTGCCATTGCACAACTCTTT
NK1.1	ATTCACAGAGGACTTTCTTTTGCTT	GCAACACTTAACTGGTGGTGAGAA
IL1α	TGCCAGAAACACCAAAACTCATC	CCGACTTTGTTCTTTGGTGGC
TNFα	TAGCTCCCAGAAAAGCAAGC	TTTTCTGGAGGGAGATGTGG
IFNγ	AGGTCAACAACCCACAGGTC	ATCAGCAGCGACTCCTTTTC
IL1β	GAAGAAGTGCCCATCCTCTG	AGCTCATATGGGTCCGACAG
CD3E	ATGCGGTGGAACACTTTCTGG	GCACGTCAACTCTACACTGGT
B220	GTTTTCGCTACATGACTGCACA	AGGTTGTCCAACTGACATCTTTC
F4/80	TTGTACGTGCAACTCAGGACT	GATCCCAGAGTGTTGATGCAA
Ly6G	CTGCCCCACTACTCTGGACAATAC	AAACCAGGCTGAACAGAAGCACCC
HGF	ATGTGGGGGACCAAACTTCTG	GGATGGCGACATGAAGCAG
TWEAK	CCGCCAGATTGGGGAATTTAC	AGTCCAAAGTAGGTTAGGAAGGG

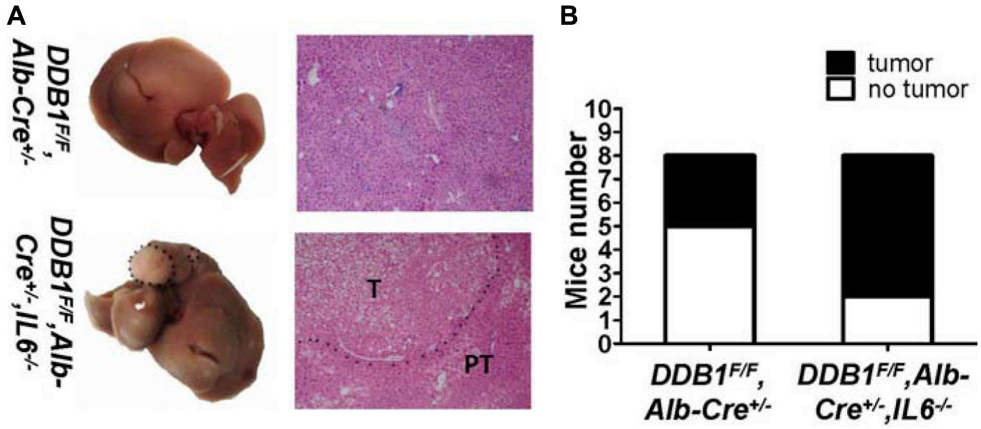

Supplementary Figure S1: Tumors arise was accelerated in IL6 deficient *DDB1$^{F/F}$,Alb-Cre$^{+/-}$* mouse. (**A**) Representative pictures of liver appearance and HE staining of liver slides(magnification,200×). (**B**) Number of mice with liver tumors at the age of 18 months.

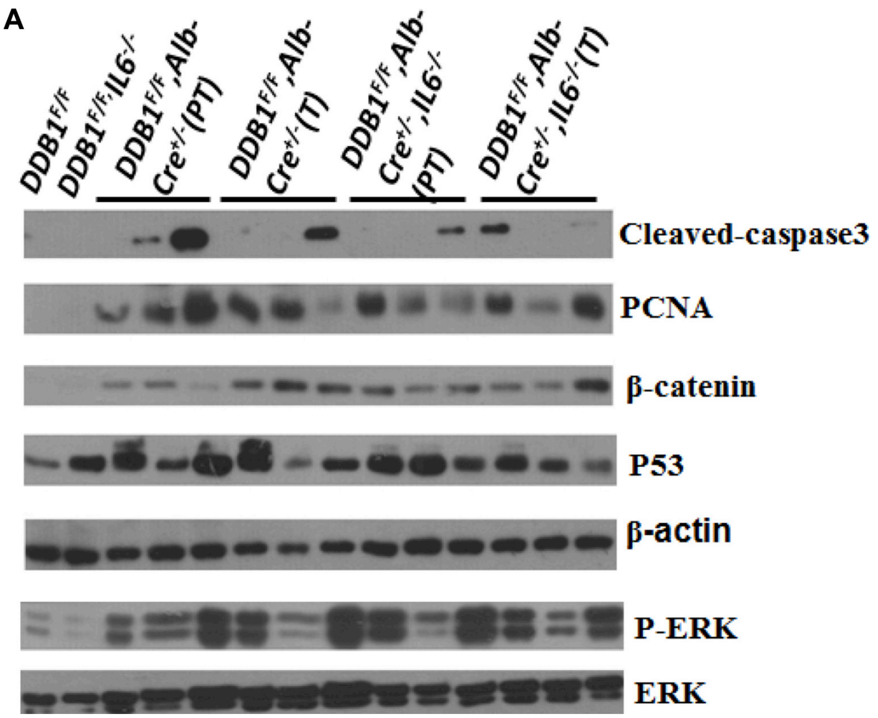

Supplementary Figure S2: No elevated cell apoptosis and compensatory proliferation in *DDB1$^{F/F}$,Alb-Cre$^{+/-}$,IL6$^{-/-}$* mouse. (**A**) Western blot for detecting cleaved-caspase3, PCNA, β-catenin, P-ERK and ERK in liver tissues of indicated mice.

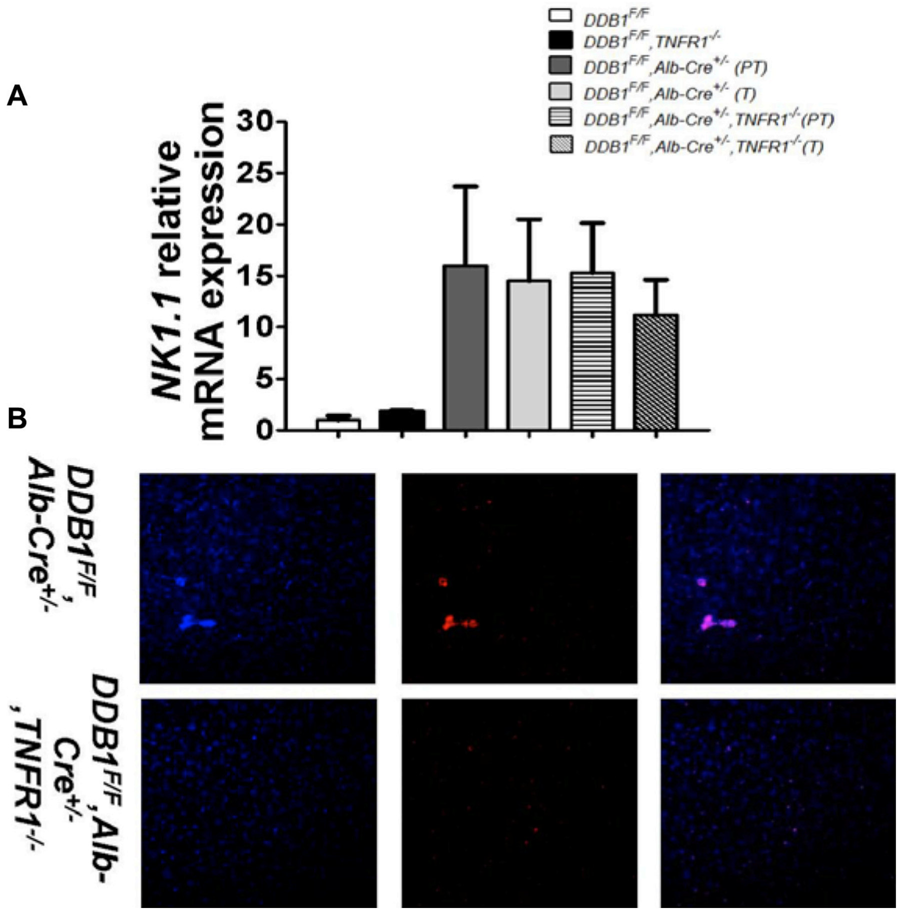

Supplementary Figure S3: TNFR1 deficiency did not affect NK cells. (**A**) The mRNA level of NK1.1 was detected by RT-PCR. Data are represented as mean ± S.E.M, n = 3–4, (**B**) IF staining for NK1.1 in tumor tissues of indicated mice. Representative pictures are shown (magnification, 200×).

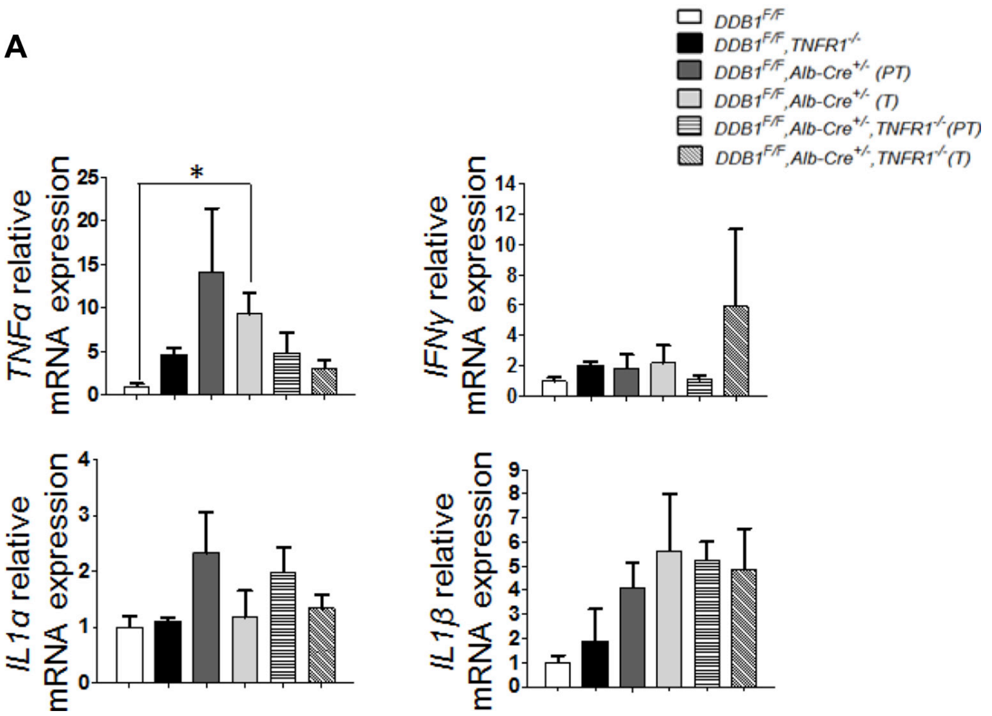

Supplementary Figure S4: Evaluated the mRNA levels of Th1-cytokines in livers of indicated mice at the age of 21 months. (**A**) the hepatic mRNA levels of TNFα,IFNγ,IL1α and IL1β were measured by RT-PCR.
